# Supplementary material for: Back Injection Molding of Sub-Micron Scale Structures on Roll-to-Roll Extrusion Coated Films
Source: Polymers (Basel). 2021 Apr 27;13(9):1410. doi: 10.3390/polym13091410 (PMC8123894; doi:10.3390/polym13091410)
Supplement: Supplementary file 1 [file polymers-13-01410-s001.zip › polymers-1193352-supplementary.pdf]

polymers

MDPI

Supplementary Material

Back Injection Molding of Sub-Micron Scale Structures on Roll-to-Roll Extrusion Coated Films

Sijia Xie [1], Jerome Werder [2] and Helmut Schift [1,*]

[1] Laboratory for Micro- and Nanotechnology, Paul Scherrer Institute (PSI), 5232 Villigen, Switzerland; sijia.xie@psi.ch
[2] Institute of Polymer Nanotechnology (INKA), FHNW University of Applied Sciences and Arts Northwestern Switzerland, 5210 Windisch, Switzerland; jerome.werder@fhnw.ch
* Correspondence: helmut.schift@psi.ch

Simulation of the film deformation with Ansys:

In order to demonstrate the effect of the geometrical dimension on the deformation of the surface structures in the film during the BIM process, we simulated and compared two structures in the PP/PET film with the analysis system "Static Structural" of the software Ansys. (Figure S1) We assumed that the thermal distribution is linear and gradually decreasing along the direction of the molding, here displayed as the Y axis, which is perpendicular to the film plane. The thermal condition in the film is set to be 200 °C at the bottom of the film and 40 °C at the top of the film where the grooves locate. We assume that the pressure (50 MPa) is applied along the Y axis, too, during the BIM process. A directional deformation can then be calculated, when the top surface of the square grooves is set as the fix support. For the two films which have a same total film thickness of 90 µm, the deformation in the grooves (a), of which period 110 µm, groove opening width 55 µm, and groove depth 30 µm, is stronger than the grooves in (b), of which period 40 µm, groove opening width 20 µm, and groove depth 25 µm. The simulation result indicates that the dimensions, such as the groove width, will affect the deformation of the structures, when the other conditions are comparable.

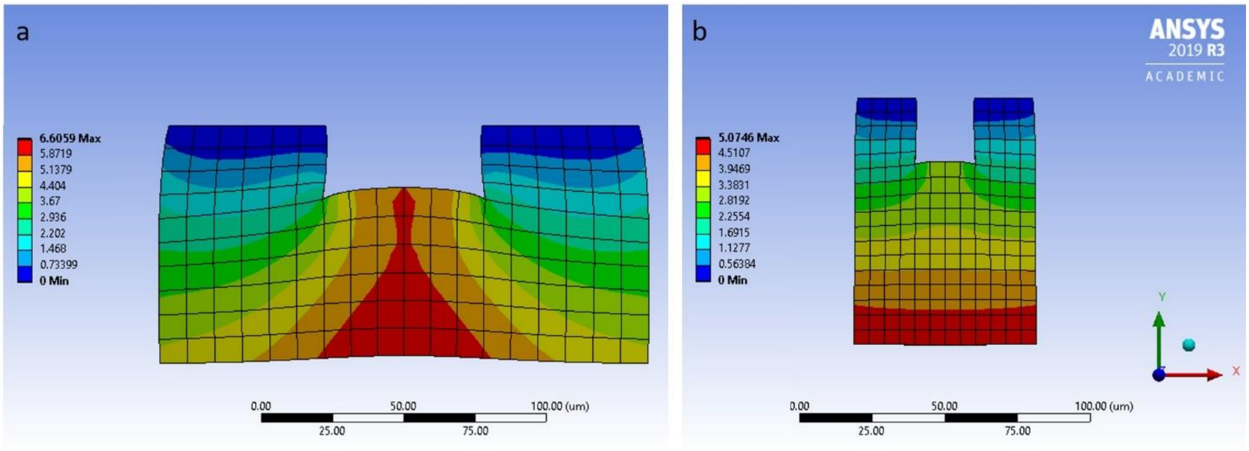

Figure S1. Modelling of the structural deformation in the 90 µm thick PP/PET films during the BIM process. (**a**) film with square grooves, period 110 µm, groove opening width 55 µm, and groove depth 30 µm. (**b**) film with square grooves, period 40 µm, groove opening width 20 µm, and groove depth 25 µm. The color scale bars indicate the level of the directional deformation.

Peel-off test:

Figure S2 shows the setup of the hand-operated peel-off test. The result of the measurement is listed in Table 3.

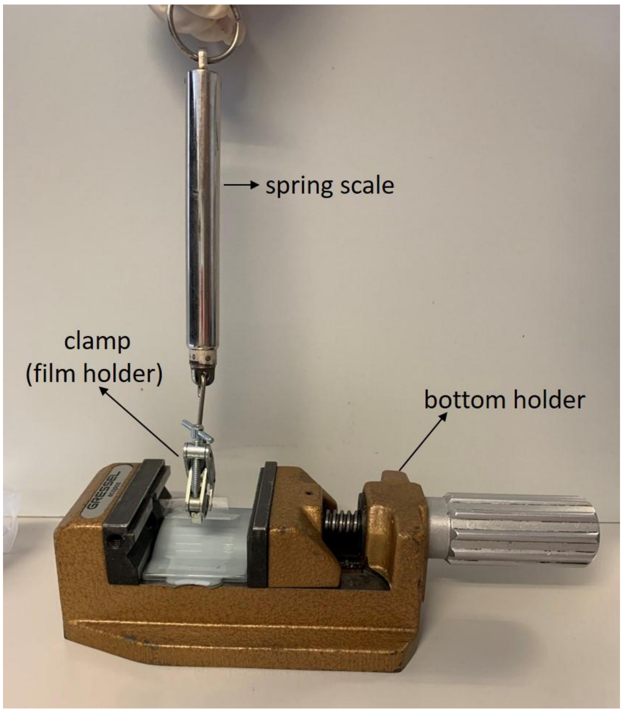

Figure S2. Photo of the hand-operated setup for the pull-off force test. The force is either calculated from the weight that is read from the spring scale, or directly read from an electronic spring scale, at the moment of delamination.
